# Peripheral venous catheter-related bloodstream infection is associated with severe complications and potential death: a retrospective observational study

**DOI:** 10.1186/s12879-017-2536-0

**Published:** 2017-06-17

**Authors:** Akihiro Sato, Itaru Nakamura, Hiroaki Fujita, Ayaka Tsukimori, Takehito Kobayashi, Shinji Fukushima, Takeshi Fujii, Tetsuya Matsumoto

**Affiliations:** 1 Department of Infection Prevention and Control, Tokyo Medical University Hospital, 6-7-1 Nishishinjuku, Shinjuku-ku, Tokyo 160-0023 Japan; 2 Department of Infection Prevention and Control, Tokyo Medical University Hachioji Medical Center, 1163 Tatemachi, Hachioujishi, Tokyo 193-0998 Japan; 3 Department of Microbiology, Tokyo Medical University, 6-1-1 Shinjuku, Shinjuku-ku, Tokyo 160-8402 Japan

**Keywords:** Peripheral venous catheter-related bloodstream infection, Catheter-related bloodstream infection, Sepsis, *Staphylococcus aureus*

## Abstract

**Background:**

The purpose of this study was to identify the clinical characteristics and outcomes of peripheral vascular catheter-related bloodstream infections (PVC-BSIs) and determine the risk of severe complications or death.

**Methods:**

We performed a retrospective observational study from June 2010 to April 2015 at two regional university-affiliated hospitals in Tokyo. We studied the clinical manifestations, underlying diseases, laboratory results, treatment methods, recurrence rates, and complications in 62 hospitalized patients diagnosed with PVC-BSIs by positive blood cultures.

**Results:**

The median time from admission to bacteremia was 17 days (range, 3–142 days) and that from catheter insertion to bacteremia diagnosis was 6 days (range, 2–15 days). Catheter insertion sites were in the arm in 48 (77.4%) patients, in the foot in 3 (4.8%) patients, and in an unrecorded location in 11 (17.7%) patients. Additionally, the causative pathogens were Gram-positive microorganisms in 58.0% of cases, Gram-negative microorganisms in 35.8% of cases, Candida spp. in 6.2% of cases, and polymicrobials in 25.8% of cases. Eight (12.9%) patients died within 30 days of their blood culture becoming positive. Patients who died of PVC-BSIs had a higher proportion of *Staphylococcus aureus* infection than patients who survived (odds ratio, 8.33; *p* = 0.004).

**Conclusions:**

PVC-BSIs are a significant cause of health care-associated infection. We observed cases of severe PVC-BSI requiring intensive and long-term care along with lengthy durations of antibiotic treatment due to hematogenous complications, and some patients died. For patients with PVC-BSIs, *S. aureus* bacteremia remains a major problem that may influence the prognosis.

## Background

Reported nosocomial infection sites include the urinary tract (34%), surgical wounds (17%), bloodstream (14%), and lower respiratory tract (13%) [1]. The catheter retained in a blood vessel is the most common cause of bloodstream infections, and catheter-related bloodstream infection (CRBSI) has been the subject of extensive surveillance and research. However, although intravenous devices are indispensable in modern-day medical practice and are used in most hospitalized patients, many such reports involve central venous CRBSIs (CVC-BSIs), not peripheral vascular CRBSIs (PVC-BSIs), and clinical and epidemiological data on PVC-BSI are lacking [2–5]. A previous study reported an incidence of 0.5 and 2.7 episodes per 1000 patient-days for PVC-BSI and CVC-BSI, respectively. The reported incidence of PVC-BSIs is lower than that of CVC-BSIs, but the cumulative duration of PVC insertion is 15 times that of CVC insertion because PVCs are inserted in 30 to 80% of inpatients [6]. Although the frequency of infection is low, the number of PVC-BSIs is high because of the high number of patients undergoing PVC insertion.

In the clinical setting, we sometimes observe PVC-BSI cases that subsequently cause local and systemic complications, suppurative thrombophlebitis, and osteomyelitis. These complications may become severe, necessitating admission to the intensive care unit and resulting in death in some cases. In particular, superficial thrombophlebitis occurs at a frequency ranging from 6.8 to 21.7% [7, 8]. Risk factors for thrombophlebitis are numerous but depend mainly on the type of infusion and duration of peripheral catheter use [9]. Local infection occurs in 0.08–6.90% of cases, but its course is usually mild [10]. Severe infection is much less frequent (0.036–0.100% of cases) but is responsible for high morbidity and mortality rates [11]. Moreover, these severe complications lengthen the treatment period and worsen the prognosis. We must, therefore, recognize that in addition to CVC-BSIs, PVC-BSIs are also important nosocomial infections.

The objective of our study was to describe the clinical characteristics and outcomes of PVC-BSIs and elucidate their associated risk of severe complications or death.

## Methods

We retrospectively studied the clinical manifestations, underlying diseases, laboratory results, treatment methods, recurrence rates, and complications in 62 patients diagnosed with PVC-BSIs by positive blood cultures from 1 June 2010 to 30 April 2015 at two regional hospitals in Tokyo: Tokyo Medical University Hospital (a 1015-bed university-affiliated hospital) and Tokyo Medical University Hachioji Medical Center (a 610-bed university-affiliated hospital). The study population included all hospitalized patients.

The diagnostic criteria for PVC-BSIs were as follows: I) growth of concordant bacterial species in a semi-quantitative tip culture and percutaneously drawn blood cultures without another apparent source of bacteremia, or II) catheter tip culture was not performed but bacteremia was present with one of the following conditions: (i) phlebitis (including redness, swelling, pain, or purulent discharge) or (ii) clear resolution of clinical symptoms upon catheter withdrawal and careful exclusion of an alternative explanation for the bacteremia. The infectious disease specialists in our hospitals personally examined all patients at the time when blood cultures began showing positive results and excluded any other apparent sources of bacteremia. Overall mortality was defined as the death of the patient within 30 days of hospitalization after the BSI.

PVCs were usually inserted by doctors and maintained by ward nurses using aseptic techniques. During the 5 years of this study, there were not any policy or protocol changes that affected the way in which PVCs were inserted. The skin was prepared with a single-patient applicator containing 76.9 vol% to 81.4 vol% ethanol and allowed to dry. The physician’s hands were decontaminated using an established hand hygiene procedure before and after contact with each patient. Hands were cleaned immediately before donning and after removing gloves. Gloves were single-use and were removed and discarded immediately after use. A sterile, transparent, semipermeable dressing was used to allow for daily observation of the insertion site. PVCs were re-sited every week or sooner if there was evidence of phlebitis.

Blood specimens were inoculated in BACTEC Culture Media (Becton, Dickinson and Company, Tokyo, Japan). Blood was cultured with the BACTEC 9240 system (Becton, Dickinson and Company), and strains were identified with the MicroScan WalkAway-96 (Beckman Coulter, Inc., Brea, CA, USA). Breakpoints of each antibiotic were in accordance with Clinical and Laboratory Standards Institute breakpoints. We calculated the percentage of each category of causative pathogen, including duplicate polymicrobials.

Statistical analysis was performed using SPSS for Windows, version 24 (IBM SPSS, Inc., Chicago, IL, USA). Mean values were compared using the two-sample *t*-test for independent samples and the chi-squared test or Fisher’s exact test as appropriate. For continuous variables, Student’s *t* test or the Mann–Whitney *U* test was performed depending on the distribution of the respective variable. Statistical significance was defined as *p* < 0.05.

## Results

The case characteristics are summarized in Table 1. The causative pathogens were Gram-positive microorganisms in 58.0%, Gram-negative microorganisms in 35.8%, Candida spp. in 6.2%, and polymicrobials in 25.8% of cases; the calculated percentages of each causative pathogen type included the duplicate polymicrobials. The 13 catheter tip cultures that were able to be analyzed became positive, but the catheter had already been removed at the time of PVC-BSI diagnosis in all 49 other cases, so those catheter tips could not be cultured. A total of 23 cases had blood cultures turn positive more than twice: 16 (25.8%) cases turned positive twice, 4 (6.5%) cases turned positive three times, and 3 (4.8%) cases turned positive five times.

**Table 1:** Comparison between patients who died within 30 days of bacteremia diagnosis and patients who survived

	Full cohort *n*(%)	Died within 30 days *n* (%)	Survived *n* (%)	Odds ratio	95% CI	*P*
Total	62	8(100)	54(100)			
Gender (male)	40 (64.5)	6 (75.0)	34 (63.0)			0.512
Age, median (years)	69 (range; 1–92)	76 (range; 52–86)	69 (range; 1–92)			0.126
Duration from admission to bacteremia, median (days)	17 (range; 3–142)	33(range; 9–142)	16(range; 3–86)			0.089
Duration of antibiotic therapy from diagnosis of PVC-BSI, median (days)	16 (range; 5–100)	17(range; 7–46)	16(range; 3–100)			0.737
Time from catheter insertion to bacteremia, median (days)	6 (range; 2–15)	7 (range; 2–9)	7 (range; 3–15)			0.526
Unknown	26 (41.9)	2 (25.0)	24 (44.4)	0.42	0.08–2.25	0.298
< 3 days	4 (11.1)	2 (25.0)	2 (3.7)	8.67	1.03–73.25	0.022
4–7 days	19 (52.8)	1(16.7)	18 (66.7)	0.29	0.03–2.50	0.232
> 8 days	13 (36.1)	3(50.0)	10 (30.0)	2.64	0.54–12.91	0.218
Causative microorganism (including duplicate polymicrobials)						
Gram-positive microorganisms	47(58.0)	7(77.8)	40(54.8)	2.45	0.28–21.72	0.408
*S.aureus*	14(17.3)	5(62.5)	9(16.7)	8.33	1.68–41.29	**0.004**
MSSA	10(71.4)	3(37.5)	7(12.7)	4.03	0.78–20.70	0.078
MRSA	4(28.6)	2(25.0)	2(3.7)	8.67	1.03–73.25	0.022
CNS	21(25.9)	2(25.0)	16(29.6)	0.79	0.14–4.35	0.788
*Bacillus* spp.	6(7.4)	0	6(11.1)	0.44	0.02–8.53	0.576
*Enterococcus* spp.	6(7.4)	0	6(11.1)	0.44	0.02–8.53	0.576
Gram-negative microorganisms	30(35.8)	2(22.2)	28(38.4)	0.31	0.06–1.67	0.156
*Enterobacter* spp.	9(11.1)	0	6(11.1)	0.44	0.02–8.53	0.576
*Klebsiella* spp.	5(6.2)	1(12.5)	4(7.4)	1.79	0.17–18.35	0.621
*Pseudomonus* spp.	4(4.9)	1(12.5)	3(5.6)	2.43	0.22–26.69	0.455
*Acinetobacter* spp.	4(4.9)	0	4(7.4)	0.66	0.03–13.40	0.785
*Serratia spp.*	4(4.9)	0	4(7.4)	0.66	0.03–13.40	0.785
*E.coli*	3(3.7)	0	3(5.6)	0.87	0.04–18.29	0.926
*Citrobacter* spp.	1(1.2)	0	1(1.9)	2.10	0.08–55.84	0.651
*Candida* spp.	5(6.2)	0(0.0)	5(6.8)	0.53	0.03–10.48	0.671
Polymicrobial	16(25.8)	1(12.5)	15(27.8)	0.37	0.04–3.28	0.357
Underlying diseases (including duplicate)						
Malignancy	33 (53.2)	4(50.0)	26(48.1)	1.08	0.24–4.76	0.922
Hypertension	14 (22.6)	0	14	0.16	0.01–0.03	0.172
Diabetes mellitus	11 (17.7)	0	11	0.22	0.01–4.15	0.274
Cerebral infarction	7 (11.3)	2(25.0)	5(9.3)	3.27	0.52–20.69	0.189
Orthopedic disorder	3 (4.8)	0	3 (5.6)	0.87	0.04–18.29	0.926
Department						
Surgery	35 (56.5)	3(37.5)	32(59.3)	0.41	0.09–1.91	0.247
Internal medicine	21 (33.9)	5(62.5)	16(29.6)	3.96	0.84–18.57	0.067
Obstetrics and Gynecology	5 (8.1)	0	5 (9.3)	0.53	0.03–10.48	0.672
Pediatrics	1 (1.6)	0	1 (1.9)	2.10	0.08–55.84	0.651
Place of insertion						
Arm	48 (77.4)	7 (87.5)	41 (75.9)	2.22	0.25–19.76	0.465
Foot	3 (4.8)	1(12.5)	2 (3.7)	3.71	0.30–46.48	0.279
Unknown	11 (17.7)	0	11 (20.4)	0.22	0.01–4.15	0.274
Complication						
Suppurative thrombophlebitis	18 (29.0)	3(37.5)	15(27.8)	1.56	0.33–7.35	0.572
Bacterial cellulitis	3 (4.8)	2 (25.0)	1 (1.8)	17.67	1.39–225.05	0.004
Osteomyelitis	2 (3.2)	0	2 (3.7)	1.24	0.05–28.02	0.894

Abbreviations: *S. aureus*, *Staphylococcus aureus*; MSSA, Methicillin-sensitive *S. aureus*; MRSA, Methicillin-resistant *S. aureus*; CNS, coagulase-negative *Staphylococcus*; *P. aeruginosa, Pseudomonus aeruginosa; E. coli, Escherichia coli*

Footnote: Some of the patients included in our study had multiple diseases. When we calculated the incidence of each underlying disease, we included the patients with multiple underlying diseases in all relevant calculations, so some patients were counted more than once

Twenty-three (37.1%) patients with complications required a longer duration of antibiotic treatment than the other 39 patients (average, 33.5 vs. 15.8 days, respectively; *p* = 0.004). All 39 of these patients had phlebitis, but only 11 underwent vascular ultrasonography. None of the patients had infective endocarditis. Nine (14.5%) patients required admission to the intensive care unit because of changes in vital signs and multiple organ failure secondary to PVC-BSIs.

Eight (12.9%) patients died within 30 days of the blood culture becoming positive (Table 2). All of these patients had an underlying immunodeficiency caused by conditions such as malignancy or leukemia.

**Table 2:** Clinical and epidemiological data of patients who died

Case no.	Time from hospitalization to bacteremia (days)	Time from catheter insertion to bacteremia (days)	Time from bacteremia to death (days)	Complication	Microbiology
1	44	Unknown	20	Suppurative thrombophlebitis	*S. aureus* *P. aeruginosa*
2	33	Unknown	12	None	CNS
3	13	2	10	Cellulitis	*S. aureus*
4	18	9	23	Cellulitis	CNS
5	9	3	5	Suppurative thrombophlebitis	*K. oxytoca*
6	44	7	19	None	*S. aureus*
7	42	8	14	None	*S. aureus*
8	142	9	25	Suppurative thrombophlebitis	*S. aureus*

Abbreviations: *S. aureus*, *Staphylococcus aureus*; CNS, coagulase-negative *Staphylococcus*; *P. aeruginosa, Pseudomonus aeruginosa; K. oxytoca*, *Klebsiella oxytoca*

We compared two groups: patients who died within 30 days of their blood culture becoming positive and patients who survived. *S. aureus* was the primary causative organism in five of the eight fatal cases. Patients who died of PVC-BSIs had a higher proportion of *S. aureus* infection than did patients who survived (odds ratio, 8.33; *p* = 0.004). A < 3-day duration from catheter insertion to bacteremia (odds ratio, 8.67; *p* = 0.022) and bacterial cellulitis (odds ratio, 17.67; *p* = 0.004) were each associated with a high 30-day mortality rate. No differences were observed in other pathogens, underlying diseases, department (surgery vs. internal medicine), or catheter insertion site (Table 1).

## Discussion

Among patients who died within 30 days, the time from catheter insertion to bacteremia ranged from 2 to 9 days, and the time from bacteremia to death ranged from 5 to 23 days. Of the 14 patients with *S. aureus* bacteremia, five (35.8%) died within 30 days of its diagnosis. We found that *S. aureus* was the pathogen most commonly associated with PVC-BSI-related death in this study (Table 1).

Catheter removal is warranted when a CRBSI is suspected. The Infectious Diseases Society of America recommends diagnosing CRBSI when the same organism is grown in at least one percutaneous blood culture and catheter tip culture [12]. However, the diagnosis of PVC-BSIs using this criterion may be difficult because the catheter has often already been removed when clinicians suspect a PVC-BSI. Only 13 (21.0%) patients underwent catheter tip culture in the present study; the catheter tips from the other 49 (79.0%) were not available to be tested. This indicates the possibility that PVC-BSIs are under-diagnosed and under-reported. We recommend culturing both the blood and catheter tip to ensure the correct diagnosis according to the IDSA guideline as soon as signs of infection, such as redness and swelling, are observed at the insertion site and the patient has a fever of unknown origin or a high C-reactive protein concentration. When two blood samples, one drawn from the catheter hub and the other from a peripheral vein, are cultured and they meet the CRBSI criteria for differential time to positivity, this can also be used for diagnosing CRBSI [12]; however, this criterion cannot be used for PVC-BSI diagnosis. Furthermore, the incidence of redness, tenderness, and pus at the catheter insertion site in patients with either type of CRBSI is only about 3% [12–14]; due to the low incidence of these symptoms, some clinicians may inadvertently overlook CRBSI. Diagnosis of PVC-BSIs is more difficult than diagnosis of CVC-BSIs for these reasons. It is important not only to examine the catheter insertion site but also to take aggressive measures to diagnose PVC-BSIs by performing blood and catheter tip cultures.

For PVC-BSI diagnosis, clinicians must both recognize the presence of PVC-BSIs and rule out other foci of infection. This study is focused only on cases in which the infections progressed to bacteremia and did not include cases with only localized infection. It is likely that the overall incidence of PVC infections is much greater than what is suggested by the number of total PVC-BSI cases in our study because the incidence of infections that remain localized is probably much greater than that of those that progress to bacteremia. It is imperative to carefully examine the insertion site daily to prevent PVC-BSIs from developing into bacteremia and to use assessment tools, such as the Visual Infusion Phlebitis score [15].

The patients in the present study were given antibiotics to treat PVC-BSIs for 5–100 days. The course of treatment, duration of antibiotics, and need for catheter removal depend on the causative organism. We recommend that primary physicians institute a therapeutic regimen according to the Infectious Diseases Society of America guidelines [12]. Patients with hematogenous complications tend to require longer durations of therapy and hospitalization. The average duration of antibiotic treatment in cases with hematogenous complications was 33.5 days, while that in cases with only phlebitis was 15.8 days (*p* = 0.004).

According to a previous study, the risk factors for hematogenous complications of *S. aureus* infection are failure to remove the catheter (relative risk, 2.28; 95% confidence interval, 1.22–4.27) and, among patients for whom the catheter has been removed, a longer mean duration from symptom onset to catheter removal (5 vs. 2 days; *p* < 0.001) [16]. The risk of increased disease severity is also higher among patients with persistent bacteremia; the interval in days between antibiotic initiation and the last positive blood culture tended to be longer in patients with delayed catheter removal than in patients with prompt removal, but this difference did not reach statistical significance in this small study (*p* = 0.07) [17]. These results might be explained in part by the failure to carefully examine the insertion site; thorough examination is imperative to prevent hematogenous complications. While there are no reports on the average cost of PVC-BSIs, treating CVC-BSIs is quite expensive and it is likely that treating PVC-BSIs infections is also costly [18, 19]. Furthermore, both the potential harm to patients and the additional costs resulting from extended hospitalization are serious problems.

With respect to microbiological data, a previous study reported that patients with PVC-BSIs had a higher proportion of *S. aureus* infections than did patients with CVC-BSIs (53% vs. 33%) [20]. When PVC-related bacteremia caused by *S. aureus* was compared with PVC-related bacteremia due to other pathogens, no differences were observed in age, sex, insertion site, time from catheter insertion to BSI, or complications.

In another study, the overall mortality rate was 29% among patients with nosocomial *S. aureus* bacteremia admitted to two hospitals in Oxford, UK [21]; however, we found a higher overall mortality rate (35.8%) among patients with *S. aureus* bacteremia in the present study. The high mortality rate observed in our study could be related to several factors, such as severe underlying disease, older age, and the presence of metastatic complications. Unfortunately, the retrospective nature of this study precluded our ability to distinguish between all-cause 30-day mortality and 30-day mortality related to PVC-BSIs.

Our study has a number of limitations, such as its retrospective nature and the lack of record concerning the infection site in 11 (17.7%) cases or the duration of catheter insertion in 26 (41.9%) cases. Furthermore, we were not able to identify cases in which only localized infection occurred at the insertion site and did not lead to bacteremia. Additional research is needed to reveal how many PVC infection cases only have a localized infection that does not lead to bacteremia. Moreover, the currently available information regarding the causative organisms of PVC-BSIs is inadequate because of a lack of surveillance data, making comparison of our data difficult. Furthermore, there is the possibility that our study had confounding factors; however, our sample size was not adequate to determine potential confounders. Future work will aim to adjust the data for the time from catheter insertion to bacteremia, department, and complications. Further investigation of these findings is warranted.

## Conclusions

PVC-BSIs are a significant cause of health care-associated infection. Here, we observed severe cases of PVC-BSI requiring intensive and long-term care along with lengthy durations of antibiotic treatment due to hematogenous complications, and some patients died. For patients with PVC-BSIs, *S. aureus* bacteremia remains a major problem that may influence the prognosis. For *S. aureus* bacteremia of unknown focus, catheter removal may prevent hematogenous complications and death. Importantly, isolated assessments of the PVC-BSI risk at medical facilities using information such as prevalence, duration of catheter insertion, and dressing management are insufficient because of a lack of surveillance data. More cases and data must be collected for further analyses on this important subject.

## Abbreviations

CRBSI: Catheter-related bloodstream infection
CVC-BSIs: Central venous catheter-related bloodstream infections
PVC-BSIs: Peripheral vascular catheter-related bloodstream infections
